# Methodological Approaches to and Reported Performance of Applications of Automated Machine Learning in Diabetes Risk Prediction: Rapid Review

**DOI:** 10.2196/87819

**Published:** 2026-05-12

**Authors:** Alexandre Castonguay, Sandrine Hegg-Deloye, Arthur Chatton, Amélie Goyette

**Affiliations:** 1 Faculté des sciences infirmières, Université de Montréal, Pavillon Marguerite d'Youville, 2375, Chemin de la Côte-Sainte-Catherine, Montréal, QC, H3T 1A8, Canada, 1 4182626594; 2 Département de médecine sociale et préventive, Université de Montréal, Montréal, QC, Canada; 3 Centre de recherche Azrieli, CHU Sainte-Justine, Montréal, QC, Canada

**Keywords:** automated machine learning, AutoML, type 2 diabetes risk prediction, multimodal data integration, machine learning validation, explainable artificial intelligence, artificial intelligence, AI

## Abstract

**Background:**

Type 2 diabetes (T2D) is a complex, chronic condition that imposes a substantial burden on health care systems. Prevention and early detection are critical to mitigating its impact. Automated machine learning (AutoML) models have the potential to predict individual risk and guide personalized interventions. However, their clinical deployment remains limited due to the retrospective nature of most datasets, a lack of external validation, and heterogeneity in variable selection.

**Objective:**

This study aimed to map AutoML approaches applied to T2D risk prediction, with a specific focus on their ability to integrate clinical, behavioral, environmental, and genomic data.

**Methods:**

A PRISMA (Preferred Reporting Items for Systematic Reviews and Meta-Analyses)-guided rapid review was conducted across 6 databases (PubMed, Scopus, Web of Science, IEEE Xplore, Google Scholar, and Embase) to identify empirical studies (published between 2015 and 2025) that used AutoML tools for T2D prediction based on at least 2 data types (eg, clinical, behavioral, environmental, and genomic). Screening, data extraction, and synthesis were performed systematically by 2 independent reviewers, with arbitration by ChatGPT acting as an artificial intelligence–based third reviewer.

**Results:**

In total, 13 studies met the inclusion criteria. Methodological diversity ranged from conventional machine learning with manual feature selection to partially or fully automated pipelines using tools such as the Tree-Based Pipeline Optimization Tool, H2O AutoML, or Azure Machine Learning. Reported performance varied (area under the curve=0.74‐0.99); however, external validation was uncommon. Behavioral and environmental data were only partially integrated, and no study incorporated genomic data despite its recognized potential. Most studies lacked transparency and reproducibility, with no public code or pipeline sharing.

**Conclusions:**

AutoML holds significant promise for improving T2D risk prediction through automation and model explainability. However, to support clinical adoption and generalizability, future AutoML pipelines must be developed using prospective, multicenter datasets; integrate diverse, harmonized data types, including genomics; and adhere to open science principles of transparency, reproducibility, and interpretability.

## Introduction

Type 2 diabetes (T2D) is a growing global public health challenge, affecting an estimated 589 million adults in 2024 (11%) and projected to reach 853 million (13%) by 2050. It is a leading cause of serious complications, such as cardiovascular disease, kidney failure, and vision loss, and is responsible for about 12% of global health expenditure [1]. Early detection through risk-based screening and targeted prevention strategies, including lifestyle interventions, is recommended by the American Diabetes Association to reduce morbidity, mortality, and the health care burden associated with T2D and its comorbidities [2]. Recent meta-analytic evidence shows that machine learning (ML) models can identify people at high risk of developing T2D with high accuracy, correctly flagging about 8 out of 10 future cases while avoiding false alarms for about 8 of 10 people who will remain diabetes-free, outperforming traditional risk scores in long-term follow-up studies [3].

Over the past decade, many studies have applied ML to predict diabetes risk using data from clinical cohorts, electronic health records, and large population studies. Comparative evaluations show that while these models can improve discrimination over traditional risk scores, such gains are often modest and vary across datasets [4]. Moreover, as seen across the broader prognostic modeling literature, calibration, defined as how closely predicted risks match actual outcomes, is rarely assessed; when evaluated, models with good discrimination have sometimes been poorly calibrated [5].

A landmark meta-analysis across diverse prognostic models found that any apparent superiority of ML over logistic regression was largely confined to studies at high risk of bias, with no advantage in those at low risk [6]. Many diabetes-related ML studies also rely on retrospective data without validation in new, independent populations, limiting confidence in their generalizability [7]. Differences in data quality, variable definitions, and study design further complicate translation into practice. To move the field forward, future work should routinely report both discrimination and calibration, assess potential clinical benefit (eg, through decision-curve analysis), and plan for robust external validation from the outset.

Automated machine learning (AutoML) frameworks aim to make the development of artificial intelligence (AI) prediction models easier and more consistent by automating key steps such as data preprocessing, feature selection, model selection, and hyperparameter tuning. Many now also include tools to help explain how the model makes predictions, such as identifying which factors are most important [8,9]. Some platforms, such as Auto-PyTorch, go a step further by automatically testing both traditional and deep learning approaches to find the best combination for the data at hand [10].

This type of standardization could improve the reproducibility and comparability of diabetes prediction studies, but real-world use still depends on strong study designs, such as testing in new patient groups and involving both data scientists and clinicians. Recent reviews point to the untapped potential of AutoML for T2D, especially for combining information from different sources, such as medical records, lifestyle factors, and genetic profiles, into more personalized risk scores [11,12]. Such capabilities are particularly relevant for T2D, where risk prediction often requires integrating heterogeneous, multimodal data spanning clinical, behavioral, and genetic domains.

At the same time, challenges remain, such as the difficulty of applying genetic risk scores across different populations and the need to make these complex models understandable to clinicians. These opportunities and barriers motivate a closer look at how AutoML is currently being used in diabetes research and whether it can truly deliver models that are both accurate and ready for clinical use.

To explore the current state of this emerging field and its practical implications, we conducted a rapid review of AutoML applications in T2D risk prediction (2015‐2025). Our aim was to summarize how these approaches have been applied, the types of data and methods used, and what has been reported about their performance and clinical readiness, thereby informing their future development and providing insights directly relevant to implementation in real-world settings.

## Methods

### Study Design

We conducted a structured rapid review following the PRISMA (Preferred Reporting Items for Systematic Reviews and Meta-Analyses) guidelines. Consistent with a rapid review approach, the study adhered to PRISMA principles but did not aim for exhaustive coverage, duplicate data extraction at all stages, or meta-analytic synthesis characteristic of a full systematic review. Methodological streamlining, including a restricted time frame and narrative synthesis, was implemented to provide a timely overview of emerging AutoML applications in a fast-evolving field. Accordingly, the findings should be interpreted as a structured mapping of methodological approaches rather than a comprehensive appraisal of the evidence. Although some features overlap with exploratory or scoping reviews, the use of predefined eligibility criteria, a focused research question, and a formal risk-of-bias assessment support the classification of this study as a rapid review. The research question was structured using the population, intervention, comparator, and outcome (PICO) framework to guide eligibility criteria, search strategy, and data extraction.

### Eligibility Criteria

Eligibility criteria were defined based on the PICO framework (Textbox 1).

Textbox 1. **Eligibility criteria, defined based on the population, intervention, comparator, and outcome (PICO) framework.**

**Population**

Studies included individuals at risk of type 2 diabetes (T2D), regardless of age, sex, or geography. Eligible datasets had to include at least one data type relevant to risk prediction—genomic, behavioral, environmental, or clinical.

**Intervention**

Eligible studies applied an AutoML framework (eg, Tree-Based Pipeline Optimization Tool, H2O AutoML, auto-sklearn, and Auto-PyTorch) to predict T2D onset or future risk. These tools had to automate key steps such as data preprocessing, variable selection, algorithm choice, hyperparameter tuning, or model ensembling. For the purpose of this review, AutoML was defined pragmatically as the use of computational pipelines that automate one or more key stages of the modeling workflow. Individual techniques, such as least absolute shrinkage and selection operator regression or gradient-boosted trees (eg, extreme gradient boosting), were considered part of an AutoML approach only when embedded within a partially or fully automated pipeline, rather than applied as standalone, manually specified models.

**Comparator**

When present, comparators included conventional machine learning workflows (manual feature selection and tuning) or alternative AutoML pipelines. Studies without comparators were also eligible.

**Outcomes**

Studies had to report how AutoML was implemented (tools, data types, and modeling strategies) and its predictive performance, including interpretability, the integration of multiple data sources, and methodological innovations for real-world use.

Eligible studies were empirical investigations using AutoML to predict T2D onset or risk from observational or experimental datasets (eg, cohorts, case-control studies, clinical datasets, or open-access data).

### Search Strategy

A structured search strategy was developed, combining four thematic blocks: (1) diabetes (“diabet*,” “type 1,” “type 2,” “T1D,” “T2D,” “pre-diabet*,” “insulin resistance,” “impaired glucose tolerance,” “HbA1c,” “hyperglycemia,” and “glycemic control”), (2) AutoML and ML (“automated machine learning,” “machine learning,” “artificial intelligence,” “deep learning,” “neural network,” “random forest,” “support vector machine,” “decision tree,” “XGBoost,” “TPOT,” “H2O.ai,” “DataRobot,” “feature selection,” and “pipeline automation”), (3) data types of interest (“genom*,” “DNA,” “SNP,” “gene expression,” “epigenetic,” and “GWAS”; “diet,” “physical activity,” “sleep,” and “lifestyle”; “pollution,” “air quality,” “urban,” “deprivation,” and “exposure”; and “combined model,” “multi-input,” and “fusion model”), and (4) prediction (“predict*,” “forecast*,” “risk model*,” “classification model*,” and “risk prediction”). This strategy was adapted and applied across 6 databases: PubMed, Scopus, Web of Science, IEEE Xplore, Google Scholar, and Embase. Filters were used to limit the search to original scientific articles published between January 2015 and May 2025 in English or French.

### Study Selection and Reference Management

All 851 retrieved studies were imported into Covidence (Veritas Health Innovation), and duplicates were removed. Title and abstract screening were performed independently by 2 reviewers, with ChatGPT (OpenAI) acting as an AI-based third reviewer for adjudication. Articles deemed potentially relevant (59/743, 7.9%) underwent full-text assessment against the predefined eligibility criteria using the same dual-reviewer process and AI-assisted adjudication.

### Data Extraction and Synthesis

Data extraction for the final 22.0% (13/59) of the studies was conducted in Covidence using a standardized form pretested on 3 to 5 studies. Extracted fields covered bibliographic details, study context, design, population, data types, predictors, AutoML implementation, performance metrics, interpretability, reproducibility, limitations, and clinical relevance. All data were cross-checked for completeness and consistency. ChatGPT served as an AI-assisted third reviewer solely to support adjudication in cases of disagreement by summarizing relevant methodological elements; final decisions were reached by consensus between human reviewers and were not determined by AI-generated outputs. All final inclusion decisions were verified and confirmed by human assessors, strengthening methodological rigor. The extracted study-level data are provided in Multimedia Appendix 1.

### Data Analysis

Extracted data were narratively synthesized and summarized in tables, detailing study methods, data types, AutoML approaches, and key performance metrics, including calibration. The analysis emphasized validation strategies, interpretability, and clinical applicability. All synthesis steps were cross-checked by ChatGPT as an AI-based third reviewer to ensure coherence. Risk of bias was assessed using the PROBAST (Prediction Model Risk of Bias Assessment Tool)+AI tool [7], evaluating participants, predictors, outcomes, and analysis domains.

## Results

### Study Selection

**Figure 1.**
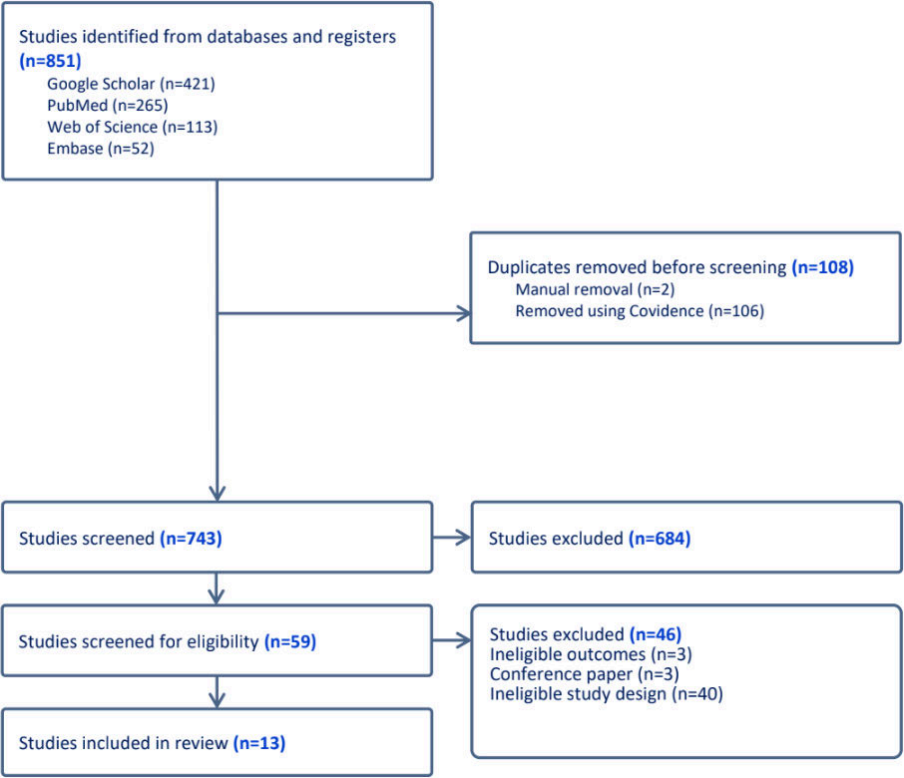
PRISMA (Preferred Reporting Items for Systematic Reviews and Meta-Analyses) flow diagram of study selection.

The database search yielded 851 records, of which 743 (87.3%) were screened. Following title and abstract screening and full-text review, 13 studies published between 2017 and 2025 met the eligibility criteria (Figure 1; Checklist 1).

The studies spanned Brazil, Greece, India, Australia, South Korea, Pakistan, China, Spain, the United States, Japan, and Iraq and used a variety of designs. Most were observational (prospective or retrospective), although 1 study conducted in 2022 by Mora-Ortiz et al [13] used a randomized controlled trial dataset. Among the 13 included studies, 2 (15.4%) used fully automated pipelines, 1 (7.7%) used semiautomated workflows, and 10 (76.9%) relied predominantly on manual ML approaches. Sample sizes ranged from 183 participants in a targeted clinical trial to 25,406 participants in a large community-based cohort.

### Scope of Outcomes

Not all studies predicted incident T2D. Several addressed related but distinct outcomes, including diagnosis, disease subtyping, or engagement in prevention programs. These outcomes differ fundamentally in clinical meaning, prediction horizon, and class balance. Consequently, performance metrics such as the area under the receiver operating characteristic curve (AUROC) or accuracy are not directly comparable across studies, and apparent differences in performance should be interpreted with caution. Therefore, AUROC values derived from these different prediction tasks should not be interpreted as directly comparable.

### Data Sources and Feature Types

Clinical variables, such as anthropometry, biochemical measures, and demographics, were the most common predictors. Behavioral variables appeared in several studies; genomic and environmental features were rare or absent. Data sources included large public cohorts (eg, Brazilian Longitudinal Study of Adult Health, English Longitudinal Study of Ageing–UK, Korean Genome and Epidemiology Study, and University of California, Irvine repositories); commercial platforms (eg, Noom); and local hospital records. The number of predictors ranged from fewer than 10 to more than 200.

### AutoML Tools and Modeling Approaches

A few studies used general-purpose AutoML frameworks (eg, Tree-Based Pipeline Optimization Tool [TPOT], H2O AutoML, and Azure Machine Learning), while many relied on partially automated or traditional workflows. Common algorithms included random forests, gradient-boosted methods, artificial neural networks, and logistic regression. Most studies combined limited automation (eg, feature selection or tuning) with manual preprocessing, corresponding to semiautomated pipelines under the operational definition used in this review.

### Validation Strategies

Internal validation was common, particularly 10-fold cross-validation and holdout splits (eg, 70/30 or 80/20), often with stratified or repeated sampling. External or prospective validation was reported in a small subset, and performance sometimes declined compared with internal results. A few studies incorporated external or prospective validation [14,15]; however, these were the exception.

### Model Performance

Reported performance varied by dataset, outcome definition, and modeling approach. AUROC values ranged from approximately 0.74 to 0.99 across studies, and accuracy ranged from approximately 75% to 97.7%. In some cases, AutoML-derived models outperformed established clinical risk scores; for instance, Fazakis et al [4] achieved an AUROC of 0.884 compared with 0.821 for the Finnish Diabetes Risk Score (FINDRISC). AUROC values near 0.99 were observed primarily in training or test splits or for nonincidence tasks (eg, engagement prediction in digital prevention programs), and these often lacked external validation [4]. However, calibration measures and decision-analytic metrics were rarely reported, limiting the clinical interpretability of high AUROC values.

### Interpretability and Reproducibility

Most studies included some form of interpretability, such as feature importance rankings, Shapley additive explanations (SHAP) values, or Boruta-based feature selection. Reproducibility was rarely addressed: few studies provided open-source code, complete model parameters, or complete datasets.

### Clinical Readiness

Although several studies discussed potential clinical applications, only a minority incorporated elements aligned with real-world implementation, such as prospective validation, workflow integration, or usability testing for clinicians. Overall, methodological performance was often high, but concrete steps toward clinical adoption remained limited.

### Risk of Bias: PROBAST Assessment

A risk-of-bias assessment using the PROBAST framework examined 4 domains: participants, predictors, outcomes, and analysis.

In the participants domain, most studies showed a high risk of bias, although populations were generally well defined and clinically relevant [16,17].

The predictors domain was more heterogeneous: some studies used automated feature selection methods such as least absolute shrinkage and selection operator (LASSO) or SHAP, while others relied on manual or poorly documented approaches, leading to moderate to high risk of bias. In the outcome domain, risk was low, as predictive targets were consistently well defined [4,14,16,18].

Regarding analysis, most studies applied appropriate validation techniques (cross-validation, training or test splits, or external validation), although a few lacked sufficient methodological detail [12,18-20].

Overall, approximately one-third of studies were at high risk of bias, one-third at moderate risk, and only a few at low risk across all PROBAST domains.

Across studies, the PROBAST domains most frequently associated with high risk of bias were predictor selection and analysis, often due to manual feature selection, limited reporting of preprocessing steps, or the lack of external validation. Studies using more automated and standardized pipelines tended to show lower risk in the predictors domain but not necessarily in the analysis domain. Notably, very high AUROC values were more frequently observed in studies with a higher overall risk of bias.

## Discussion

### Principal Findings

In this review, clinical readiness was defined as the presence of key elements required for real-world deployment, including external validation, calibration reporting, assessment of clinical utility, workflow integration, and interpretability for end users. The included studies spanned multiple regions and addressed heterogeneous objectives, such as early screening, cardiovascular risk stratification, T2D subtyping, and support for digital prevention programs [13,16-18]. This diversity reflects the breadth of current AutoML applications in diabetes research but also underscores the need for caution when comparing performance across fundamentally different prediction tasks.

From a methodological standpoint, supervised algorithms such as random forest, extreme gradient boosting (XGBoost), and light gradient boosting machine were predominant, balancing performance and interpretability. Several studies used SHAP or decision trees to identify key predictors and improve clinical usability [4,14]. However, most relied on retrospective, single-center data, with limited external validation and potential selection bias [12,14,16]. This gap between methodological rigor and generalizability remains a major barrier to clinical translation.

Bias also arose from manual predictor selection [16,18]. In contrast, studies embedding techniques such as LASSO or Boruta within automated or semiautomated pipelines demonstrated improved standardization [4,14,17]. In this review, such techniques were considered part of AutoML only when integrated into an automated workflow rather than applied as standalone methods. Fully automated AutoML pipelines—encompassing preprocessing, feature selection, and hyperparameter tuning—remained rare; only 1 study implemented Azure Machine Learning without complete pipeline automation [19]. Nevertheless, a gradual shift toward semiautomated and externally validated approaches was observed over time [12,14].

Reported performance was high (area under the curve=0.74-0.99), but comparability suffered from heterogeneous outcomes and metrics [13,18]. Few studies reported calibration or predictive value measures, despite their importance for clinical risk prediction. Reproducibility also remained a major weakness, with no study sharing full code or modeling pipelines, contrary to open science recommendations [4,14]. Overall, AutoML approaches did not consistently outperform non-AutoML models under rigorous validation conditions, underscoring the primacy of external validation and calibration over nominal performance gains, particularly across heterogeneous prediction tasks.

Finally, interpretability remains a key challenge, as AutoML systems are often perceived as “black boxes” [11]. Integrating explainable AI tools such as SHAP, local interpretable model-agnostic explanations, and integrated gradients directly into AutoML frameworks (eg, AutoGluon) could enhance transparency and usability, thereby supporting clinical adoption [11]. Future research should prioritize data diversity; prospective multicenter validation; and the development of standardized, shareable AutoML pipelines to facilitate reproducibility and real-world implementation [4,12,14].

Notably, none of the reviewed studies included genomic data, despite its potential to improve prediction accuracy. Beyond technical considerations, practical barriers such as cost, limited availability of genomic data in routine care, population-specific transferability of polygenic risk scores, and data governance constraints likely contribute to the absence of genomic predictors in current AutoML-based T2D models. Nevertheless, existing evidence suggests that integrating polygenic risk information could enhance future AutoML-based prediction models [17].

### Reported Limitations and Author Suggestions

The included studies reported several recurring limitations, notably the reliance on regional or hospital-based datasets, limited external or prospective validation, and incomplete integration of behavioral or genomic predictors. Authors consistently emphasized the need for broader and more diverse predictor sets, multicenter study designs, and prospective validation. Taken together, these limitations mirror the methodological and clinical gaps identified in this review and underscore the need for standardized, explainable, and reproducible AutoML pipelines oriented toward real-world clinical use.

### Conclusions

This rapid review underscores the promising yet underrealized potential of AutoML for predicting T2D risk. Across 13 studies (2017‐2025), models showed strong discrimination and a growing use of interpretable methods (eg, SHAP and decision trees). However, clinical translation remains constrained by local or retrospective data; limited external validation; and the absence of fully automated, reproducible pipelines.

To overcome these gaps, future research should adopt prospective, multicenter designs and integrate diverse data sources—clinical, behavioral, environmental, and omics data. Developing standardized, transparent, and shareable AutoML frameworks is essential for building trust and enabling real-world applications.

Some actionable recommendations are as follows:

- Ensure open access to code, trained models, and reproducible pipelines
- Promote multicenter, demographically diverse cohorts, explicitly including genomic and other omic features
- Benchmark explainable AutoML frameworks (eg, SHAP integrated with AutoGluon) under prospective, externally validated conditions, with calibration and clinical utility metrics reported alongside AUROC

Collectively, these actions can transform AutoML from a high-performing research tool into a transparent, reliable decision support system with tangible public health benefits.

## Supplementary material

10.2196/87819 Multimedia Appendix 1 Extraction study-level data.

10.2196/87819 Checklist 1 PRISMA-ScR checklist.

## Abbreviations

AI: artificial intelligence
AUROC: area under the receiver operating characteristic curve
AutoML: automated machine learning
FINDRISC: Finnish Diabetes Risk Score
LASSO: least absolute shrinkage and selection operator
ML: machine learning
PICO: population, intervention, comparator, and outcome
PRISMA: Preferred Reporting Items for Systematic Reviews and Meta-Analyses
PRISMA-ScR: Preferred Reporting Items for Systematic Reviews and Meta-Analyses extension for Scoping Reviews
PROBAST: Prediction Model Risk of Bias Assessment Tool
SHAP: Shapley additive explanations
T2D: type 2 diabetes
TPOT: Tree-Based Pipeline Optimization Tool
XGBoost: extreme gradient boosting
